# Acute intermittent porphyria presenting as posterior reversible encephalopathy syndrome: a case report

**DOI:** 10.1186/s13256-026-06000-3

**Published:** 2026-04-06

**Authors:** Akashdeep Sharma, Yamini Chawla, Virendra Atam, Sagar Srivastava

**Affiliations:** 1 Internal Medicine, King George’s Medical University, Lucknow, India; 2 Sanjay Gandhi Post Graduate Institute of Medical Sciences, Lucknow, India

**Keywords:** Acute intermittent porphyria, Posterior reversible encephalopathy syndrome, Seizures, Hyponatremia, Abdominal pain, Porphobilinogen

## Abstract

**Background:**

Acute intermittent porphyria (AIP) is a rare metabolic disorder of heme biosynthesis. It usually presents with abdominal pain, gastrointestinal disturbances, and neuropsychiatric manifestations. Posterior reversible encephalopathy syndrome (PRES), characterized by seizures, altered mental status, and characteristic imaging findings, is an unusual and under-recognized presentation of AIP.

**Case presentation:**

We present the case of a 16-year-old South Asian male with a 2-month history of recurrent abdominal pain, nausea, vomiting, and constipation, who developed sudden-onset generalized tonic–clonic seizures. Initial workup revealed hyponatremia and elevated liver enzymes. MRI of the brain showed findings consistent with PRES. Further history and investigations, including elevated urinary porphobilinogen levels, confirmed the diagnosis of AIP. The patient was managed conservatively with carbohydrate loading, avoidance of precipitating factors, and antiepileptic therapy, leading to gradual clinical improvement. A repeat MRI showed partial resolution of lesions, confirming the reversibility of PRES.

**Conclusion:**

This case highlights the importance of considering AIP in young patients presenting with gastrointestinal and neurological symptoms. PRES as a manifestation of AIP is rare but should be recognized promptly, as early diagnosis and conservative management can lead to a favorable outcome.

**Supplementary Information:**

The online version contains supplementary material available at 10.1186/s13256-026-06000-3.

## Background

Porphyrias are a heterogeneous group of disorders of heme biosynthesis in which the overproduction of heme precursors, due to partial deficiency of one of the seven enzymes in the pathway, leads to characteristic clinical and biochemical features. Because porphyrias present with diverse symptoms that often overlap with other disorders, they are frequently misdiagnosed or diagnosed late. Accurate diagnosis depends on identifying specific patterns of heme precursor accumulation.

There are seven main types of porphyrias, categorized according to their clinical manifestations into neuropsychiatric, dermatological, and mixed forms [1].

Porphyrias presenting with neurological symptoms include:

1. Hereditary coproporphyria (HC)
2. Acute intermittent porphyria (AIP)
3. Variegate porphyria (VP)
4. These are typically inherited in an autosomal dominant pattern [2].

AIP results from a deficiency of hydroxymethylbilane synthase (HMBS) and presents with abdominal pain, vomiting, nausea, peripheral neuropathy, and seizures [3]. It is the most common inherited hepatic porphyria and usually affects women of reproductive age, although it can also occur in males [4]. While abdominal pain is the most common manifestation, seizures may occasionally be the presenting feature [5].

We report a case of a 16-year-old male presenting with gastrointestinal symptoms followed by sudden-onset generalized tonic–clonic seizures (GTCS). The purpose of this report is to discuss the potential differential diagnoses and diagnostic approach in such a case, emphasizing the need for a systematic evaluation of both gastrointestinal and neurological systems in young patients.

## Case presentation

A 16-year-old South Asian male presented to the medicine emergency trauma center with complaints of recurrent abdominal pain, nausea, vomiting, and constipation for the past 2 months. The abdominal pain was described as crampy and intermittent, often localized to the periumbilical region. Nausea and vomiting occurred in association with the pain, without any specific pattern or triggers. He also reported constipation during this period but denied diarrhea, hematochezia, or significant weight loss. These symptoms had been intermittent, with periods of partial relief.

He had undergone multiple investigations at peripheral private clinics, including abdominal ultrasound, CT abdomen, upper gastrointestinal endoscopy, and colonoscopy, none of which revealed significant findings.

Over the preceding 48 hours, the patient developed three episodes of generalized tonic–clonic seizures (GTCS). Each episode lasted 1–2 minutes and was followed by postictal confusion and drowsiness. There was no prior history of seizures, neurological illness, or head trauma. Family history was non-contributory, with no similar illness in siblings or relatives. The patient was a school student, non-smoker, and non-alcoholic, with a supportive family background and no psychosocial stressors. During inpatient care, it was observed that episodes of abdominal pain were often associated with fasting.

At admission, his vital signs were as follows: blood pressure—156/88 mmHg, pulse rate—110 beats per minute, respiratory rate—16 breaths per minute, and SpO₂—98% on room air. General physical examination was unremarkable. Neurological examination revealed normal tone and power, with no focal deficits or cranial nerve involvement. Blood pressure and pulse rate fluctuated during hospitalization, remaining elevated most of the time.

Routine laboratory investigations showed hyponatremia (serum sodium: 127 mmol/L) and elevated liver enzymes (SGOT 73 IU/L, SGPT 205 IU/L, ALP 283 IU/L). Serum amylase and lipase were within normal limits. Testing for lead toxicity showed normal results. Cerebrospinal fluid (CSF) analysis was normal. Further evaluation including autoimmune vasculitis panel and CT abdomen (see Supplementary File 1) did not reveal any significant abnormalities. Normal CSF findings, normal inflammatory markers (CRP, ESR), and normal autoimmune vasculitis panel helped rule out infective, inflammatory, or vasculitis causes. Laboratory findings are summarized in Table 1.

**Table 1:** Laboratory investigations

Parameter	Result	Reference range	Unit
Complete blood count			
Hemoglobin (Hb)	12.2	13.5–17.5 (M)/12–15.5 (F)	g/dL
Total leukocyte count	5600	4000–11,000	mm^3^
Neutrophils (%)	76	40–70	%
Lymphocytes (%)	18	20–45	%
Eosinophils (%)	2	1–6	%
Monocytes (%)	3	2–8	%
Basophils (%)	1	0–1	%
Platelet count	2.1 × 10^5^/mm^3^	1.5–4.5 × 10^5^	mm^3^
CRP	3	0–6	mg/dl
ESR	10	0–15	mm/h
Liver function tests			
SGOT (AST)	73	10–40	U/L
SGPT (ALT)	205	7–56	U/L
Alkaline phosphatase	283	40–129	U/L
Total protein	6.8	6.3–8.2	g/dL
Albumin	3.5	3.5–5.0	g/dL
Kidney function tests			
Urea	34	15–45	mg/dL
Creatinine	0.9	0.6–1.3	mg/dL
Uric acid	5.4	3.5–7.2 (M)	mg/dL
Electrolytes			
Sodium (Na⁺)	127	135–145	mmol/L
Potassium (K⁺)	3.5	3.5–5.1	mmol/L
Chloride (Cl⁻)	98	98–107	mmol/L
Bicarbonate (HCO₃⁻)	21	22–28	mmol/L
Calcium (Ca^2⁺^)	8.2	8.5–10.5	mg/dL

**Fig. 1:**
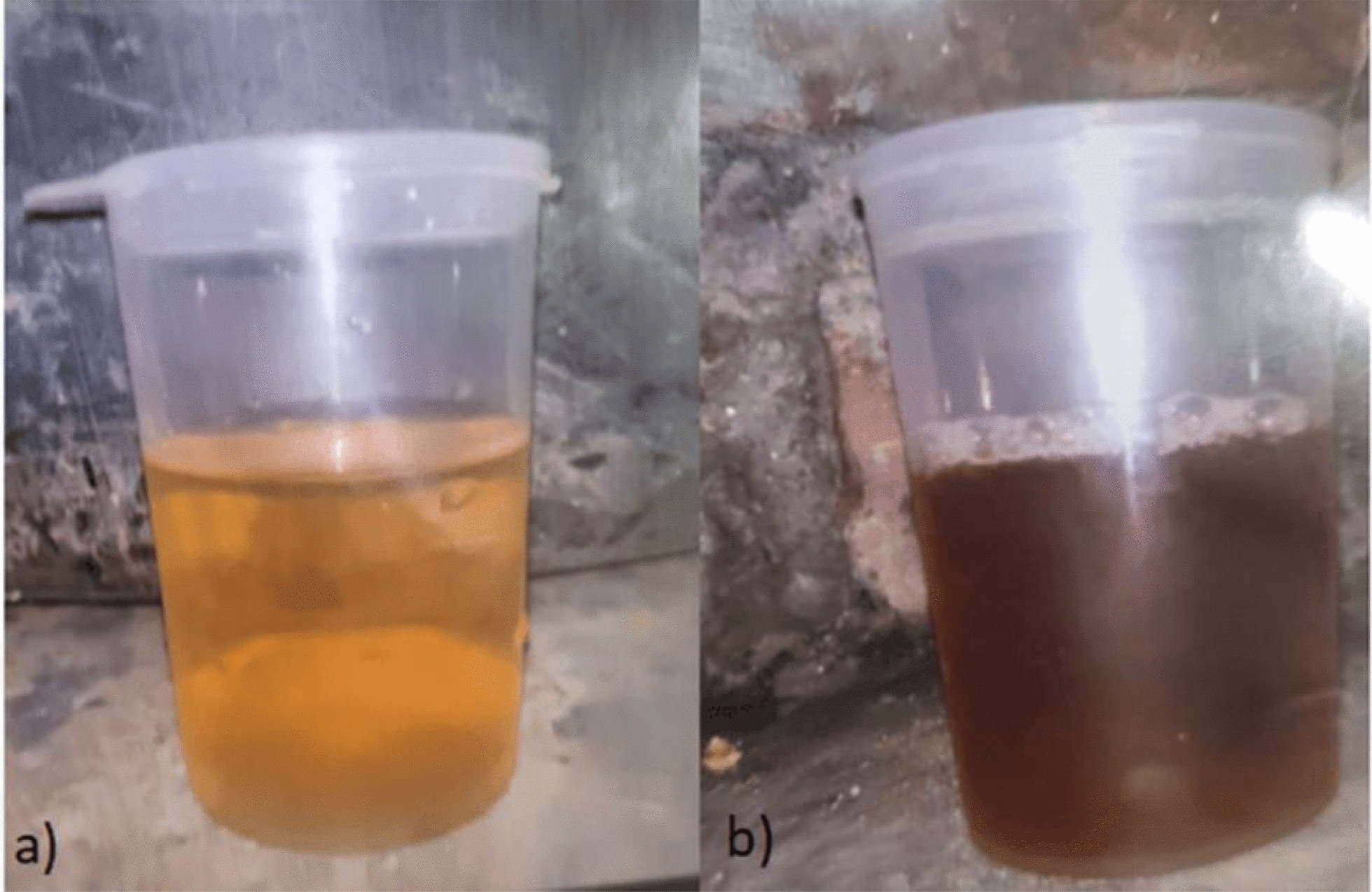
Shows darkening of urine after keeping for 24 hours in direct sunlight as described. **a** Normal color of the patient’s urine at the time of collection. **b** Darkened color of the urine observed after exposure to direct sunlight for 24 hours, indicating photosensitivity of urine pigments. This discoloration is suggestive of the presence of porphyrin precursors such as porphobilinogen and uroporphyrin, which undergo oxidation upon light exposure—a characteristic finding in disorders like acute intermittent porphyria (AIP)

The urine sample showed a characteristic darkening upon exposure to direct sunlight for 24 hours (Fig. 1), consistent with acute intermittent porphyria. Acute intermittent porphyria was confirmed by three positive tests for urinary porphobilinogen. The unavailability of genetic testing represented a resource limitation, but clinical and biochemical findings confirmed the diagnosis.

The patient’s timeline of clinical events is shown in Table 2.

**Table 2:** Timeline of clinical events

Time (T)	Clinical event/investigation	Findings/actions taken
*T* = 0 (initial presentation)	Patient presented to emergency department with 2-month history of recurrent crampy abdominal pain, nausea, vomiting, and constipation	On examination: elevated BP(156/88 mmHg) and tachycardia. Routine labs showed hyponatremia (Na⁺ 127 mmol/L), elevated liver enzymes
*T* = 0 + 1 day	Onset of episodes of generalized tonic–clonic seizures (GTCS)	Anti-epileptics started and initiated evaluation of secondary causes
*T* = 0 + 2 days	Diagnostic workup including MRI brain, CSF analysis, and lead level testing	MRI consistent with PRES; CSF and lead levels normal
*T* = 0 + 4–6 days	Urine porphobilinogen (PBG) test ordered	PBG positive on three separate tests → confirmed Acute Intermittent Porphyria (AIP)
*T* = 0 + 5 days	Nerve conduction study performed	Motor axonal neuropathy detected in bilateral ulnar, peroneal, and tibial nerves
*T* = 0 + 1 week	Initiated conservative management (high-carbohydrate diet, IV dextrose, and antiepileptics)	Clinical stabilization, reduced seizure frequency, improved abdominal pain
*T* = 2 weeks	Repeat MRI brain	Partial resolution of T2/FLAIR hyperintensities consistent with reversible pathology (PRES)
*T* = 2 months (outpatient follow-up)	Follow-up in OPD	Patient asymptomatic, no new neurological or abdominal complaints

**Fig. 2:**
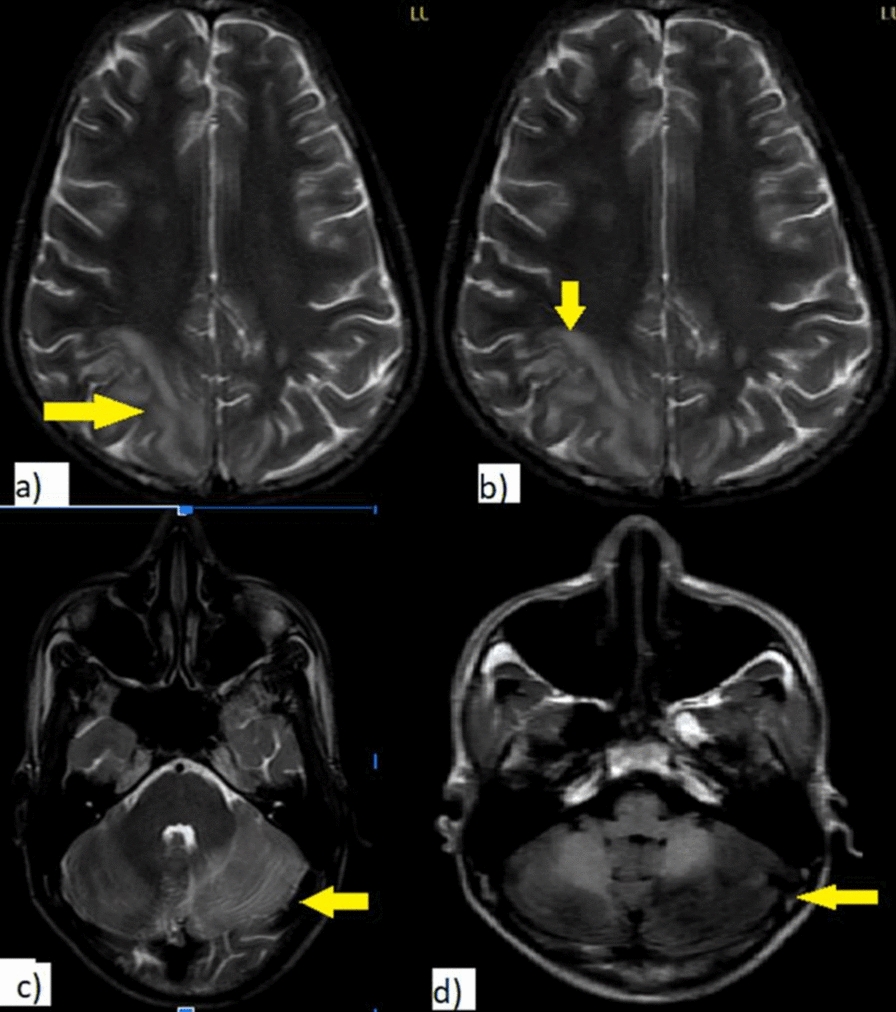
T2WI and T1WI axial images showing signal intensity of lesion as described. The T2WI axial shows hyperintensity in bilateral frontal and posterior parietal white matter (yellow arrow) (Image **a**, **b**) and left cerebellar hemisphere (Image **c**). And T1 WI (Image **d**) shows corresponding T1 iso intensity (yellow arrow) in left cerebellar hemisphere

**Fig. 3:**
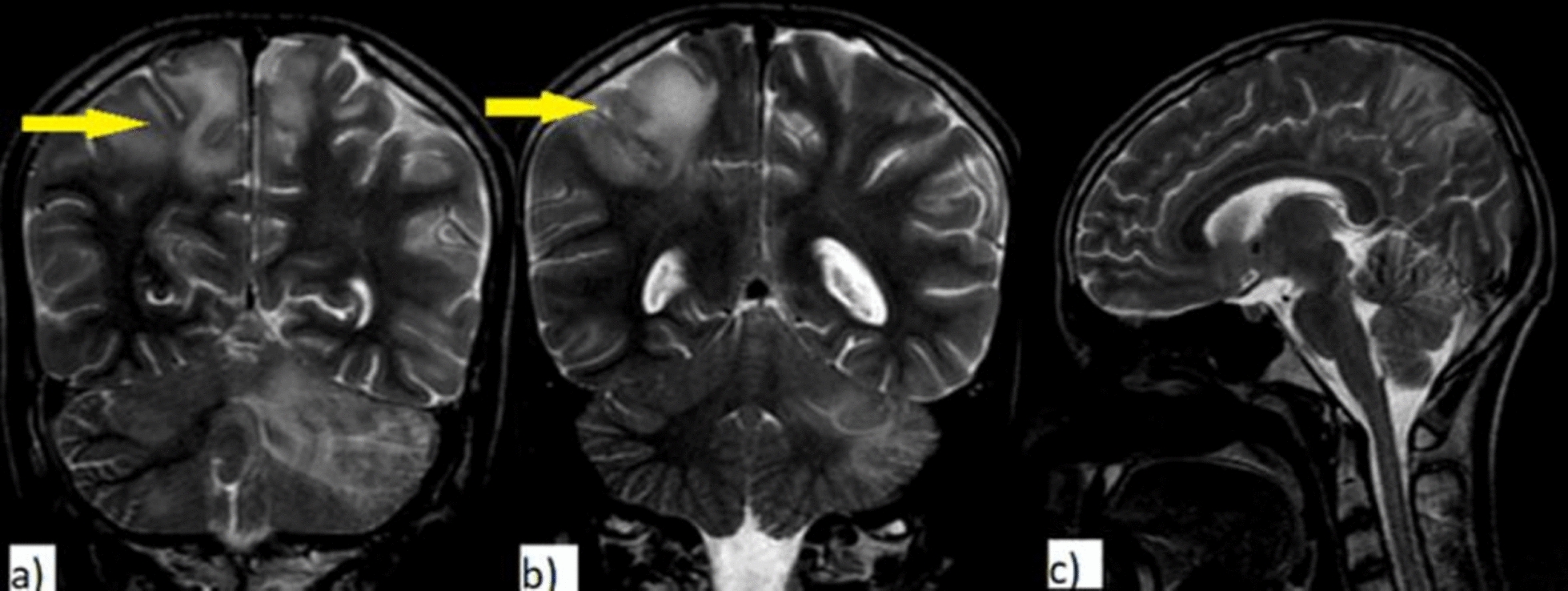
Coronal and sagittal T2WI of the same patient as described. T2WI coronal shows hyperintensity (yellow arrows) extending up to subcortical white matter (image **a**, **b**); sagittal image (image **c**) shows no involvement of corpus callosum, making MS less likely

MRI of the brain (Figs. 2 and 3) showed bilateral asymmetrical patchy T2/FLAIR hyperintensities involving the fronto-parieto-temporal regions and the left cerebellar hemisphere, without significant blooming on SWI and without post-contrast enhancement. On DWI images, focal patchy areas of true diffusion restriction were noted in the right parietal subcortical region and a small focus in the left centrum semiovale, findings consistent with posterior reversible encephalopathy syndrome (PRES).

Nerve conduction study was performed after the patient began complaining of neck pain and tingling sensations in all four limbs during admission. The study revealed motor axonal neuropathy involving the bilateral ulnar, common peroneal, and tibial nerves.

The patient was managed conservatively with dextrose-containing intravenous fluids and a high-carbohydrate diet, as hematin was not available at our center. High-carbohydrate intake suppresses hepatic ALA synthase activity, thereby reducing the accumulation of porphyrin precursors.

Hyponatremia was managed by hypertonic fluids under close monitoring of serum sodium levels and sodium levels were gradually normalized within 48–72 hours. Antiepileptic therapy with lorazepam, levetiracetam, and lacosamide was initiated, as seizures did not initially respond to monotherapy. All drugs contraindicated in acute intermittent porphyria were withheld, and prolonged fasting was avoided. The patient responded well, with a reduction in the frequency and severity of abdominal pain and cessation of seizures.

**Fig. 4:**
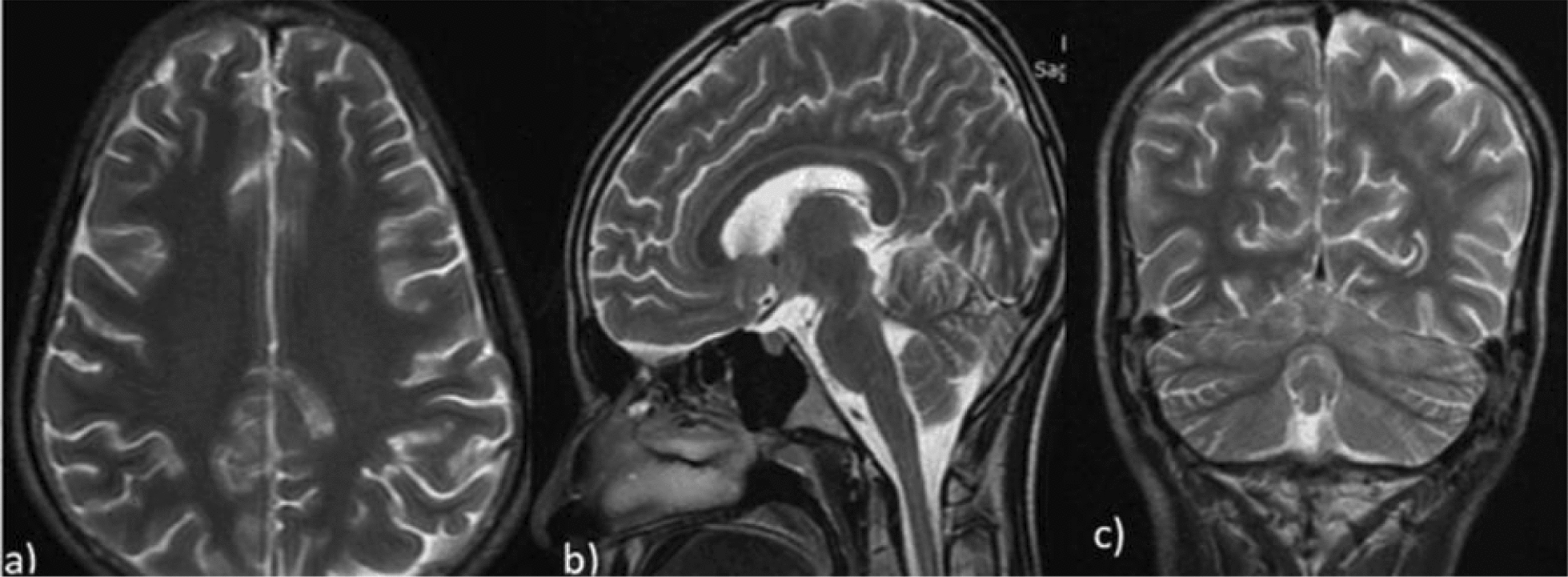
T2WI axial, sagittal, and coronal MRI shows partial resolution of lesions in the same patient after 2 weeks. Axial (image **a**), sagittal (image **b**), and coronal (image **c**) T2-weighted MRI images obtained after 2 weeks demonstrate partial resolution of previously noted hyperintense lesions consistent with posterior reversible encephalopathy syndrome (PRES), indicating partial radiological improvement in the same patient

Repeat MRI of the brain, performed 12 days after the initial scan, showed partial resolution of previous lesions (Fig. 4), consistent with a reversible pathology. These findings further supported the diagnosis of posterior reversible encephalopathy syndrome (PRES).

The patient was discharged with no new complaints and continues to remain asymptomatic on subsequent follow-up visits at 15 days, 1 month, and 2 months with no recurrence of neurological or abdominal symptoms.

## Discussion

AIP is a genetic disorder of heme biosynthesis resulting from insufficient activity of the enzyme hydroxymethylbilane synthase (HMBS) [3]. Symptoms of acute intermittent porphyria typically begin during adolescence, with a higher incidence observed in females. Acute attacks may be triggered by several factors such as fasting, certain medications, luteal-phase hormonal shifts, and alcohol intake, which increase porphobilinogen and aminolevulinic acid levels through enhanced expression of ALA synthase (ALAS1) [6]. The condition commonly presents with a combination of neurological, gastrointestinal, and autonomic symptoms. Unusual or atypical presentations can make diagnosis challenging and may delay appropriate treatment [7].

In our case, the patient initially presented with classic abdominal symptoms such as crampy pain, nausea, vomiting, and constipation, which are frequently seen in AIP. However, the diagnostic challenge arose when he developed new-onset seizures without any prior history or evident metabolic derangement, apart from hyponatremia. Neuroimaging revealed findings consistent with posterior reversible encephalopathy syndrome (PRES), which is an uncommon manifestation. By 2018, a total of 22 cases of PRES associated with AIP had been documented in the literature, as reported by Zheng et al. in their comprehensive review [8].

PRES is a clinico-radiological condition caused by vasogenic edema, usually affecting the posterior regions of the brain. Common etiologies include hypertensive crises, renal dysfunction, eclampsia, and cytotoxic drug exposure.

Peripheral neuropathy, characterized by paresthesia and motor weakness, is the most frequent neurological manifestation of AIP. Seizures may occur in both AIP and PRES [8], and they are the most common presenting feature of PRES in AIP [9].

The exact mechanism underlying PRES in AIP remains unclear, but several hypotheses have been proposed. One suggests that blood pressure elevation during an acute episode may exceed the cerebral autoregulatory limit (mean arterial pressure of 150–160 mm Hg), leading to hyperperfusion injury and subsequent PRES [10]. However, this mechanism is unlikely in our case, as the patient’s blood pressure did not exceed the autoregulatory threshold.

Another proposed mechanism involves severe heme deficiency during an acute attack, which may impair the synthesis of nitric oxide synthase (NOS), a heme-dependent enzyme. A deficiency of NOS reduces nitric oxide (NO) production, resulting in vasoconstriction and cerebral hypoperfusion. This may contribute to the development of vasogenic edema, a hallmark feature of PRES [10].

In addition to central and peripheral nervous system involvement, the autonomic nervous system may also be affected, manifesting as tachycardia, hypertension, tremors, and diaphoresis [8].

Management of acute attacks of AIP includes intravenous heme administration and carbohydrate loading if heme is unavailable. Prevention involves the avoidance of precipitating factors, and liver transplantation remains the definitive treatment option [11].

Recent literature has also reported similar cases of AIP presenting with PRES, underscoring the diagnostic challenges and management implications in resource-limited settings [12].

## Conclusion

This case demonstrates the importance of considering acute intermittent porphyria (AIP) in the differential diagnosis of adolescents presenting with unexplained gastrointestinal symptoms and new-onset seizures. The co-occurrence of AIP with posterior reversible encephalopathy syndrome (PRES), although rare, is a clinically significant manifestation that demands prompt recognition. Early identification through clinical suspicion, appropriate biochemical testing, and neuroimaging is essential to initiate timely management and avoid complications. Supportive therapy, high-carbohydrate intake, and avoidance of triggering agents remain the cornerstone of treatment if hematin is not available. Raising clinical awareness of such atypical presentations is crucial to ensuring accurate diagnosis and effective management.

## Supplementary Information

Additional file 1.

## Data Availability

The manuscript and additional document contain all the required data.

## Abbreviations

AIP: Acute Intermittent Porphyria
ALAS1: Aminolevulinic Acid Synthase 1
ALP: Alkaline phosphatase
ALT (SGPT): Alanine aminotransferase (serum glutamic-pyruvic transaminase)
AST (SGOT): Aspartate aminotransferase (serum glutamic-oxaloacetic transaminase)
CBC: Complete blood count
CECT: Contrast-enhanced computed tomography
CSF: Cerebrospinal fluid
FLAIR: Fluid-attenuated inversion recovery
GTCS: Generalized tonic–clonic seizures
Hb: Hemoglobin
HMBS: Hydroxymethylbilane synthase
KFT: Kidney function test
MCV: Mean corpuscular volume
MCH: Mean corpuscular hemoglobin
MRI: Magnetic resonance imaging
NO: Nitric oxide
NOS: Nitric oxide synthase
OPD: Outpatient department
PCV: Packed cell volume
PRES: Posterior reversible encephalopathy syndrome
SGOT: Serum glutamic-oxaloacetic transaminase
SGPT: Serum glutamic-pyruvic transaminase
SpO₂: Peripheral capillary oxygen saturation
SWI: Susceptibility weighted imaging
T1WI/T2WI: T1-weighted Imaging/T2-weighted Imaging
