# Supplementary material for: Acute intermittent porphyria presenting as posterior reversible encephalopathy syndrome: a case report
Source: J Med Case Rep. 2026 Apr 6;20:278. doi: 10.1186/s13256-026-06000-3 (PMC13188283; doi:10.1186/s13256-026-06000-3)
Supplement: Supplementary file 1 — Additional file 1. [file 13256_2026_6000_MOESM1_ESM.docx]

# **Laboratory Investigations**

## **1. Autoimmune Panel:**

ANA (Antinuclear Antibody): Negative

C-ANCA (Cytoplasmic ANCA): Negative

P-ANCA (Perinuclear ANCA): Negative

Interpretation: No serological evidence of autoimmune vasculitis or connective tissue disease.

# **Radiological Investigation**

## **Contrast-Enhanced CT (CECT) Abdomen:**

Findings:
 - No evidence of bowel wall thickening, mass, or lymphadenopathy.
 - Moderate gaseous distension of the small and large bowel loops without air-fluid levels.
 - No signs of perforation or obstruction.
 - Solid organs (liver, spleen, kidneys, pancreas) appear normal in size and attenuation.
 - No free fluid or collection noted in the abdominal cavity.

Impression:
 - Gaseous distension of bowel loops—likely ileus or early obstruction. Recommend correlation with clinical and further management accordingly.
